# Evaluation of Predisposing Factors of Necrotic Enteritis in Experimentally Challenged Broiler Chickens

**DOI:** 10.3390/ani12151880

**Published:** 2022-07-22

**Authors:** Larissa Justino, Ana A. S. Baptista, Marielen de Souza, Maísa F. Menck-Costa, Bárbara G. Pires, Claudineia E. Cicero, Ana P. F. R. L. Bracarense, Vanessa M. Kaneko, Alexandre Oba, Adriano S. Okamoto, Raphael L. Andreatti Filho

**Affiliations:** 1 Laboratory of Ornithopathology, Department of the of Veterinary Clinic, Center for Animal Pathology, School of Veterinary Medicine and Animal Science (FMVZ), São Paulo State University (UNESP), Botucatu 18618-681, Brazil; larissa.justino@unesp.br (L.J.); adriano.okamoto@unesp.br (A.S.O.); 2 Laboratory of Avian Medicine, Department of Preventive Veterinary Medicine, Center for Agrarian Sciences, State University of Londrina, Londrina 86057-970, Brazil; anaangelita@uel.br (A.A.S.B.); marielen.souza@uel.br (M.d.S.); claudineia.emidio@uel.br (C.E.C.); vanessa.kaneko@uel.br (V.M.K.); 3 Laboratory of Basic and Applied Bacteriology, Department of Microbiology, Center of Biological Sciences, State University of Londrina, Londrina 86057-970, Brazil; maisa.menckcosta@uel.br; 4 Laboratory of Animal Pathology, Department of Preventive Veterinary Medicine, State University of Londrina, Londrina 86057-970, Brazil; barbara.giglio@uel.br (B.G.P.); anapaula@uel.br (A.P.F.R.L.B.); 5 Laboratory of Animal Nutrition Agricultural Sciences Center, Department of Zootechny, State University of Londrina, Londrina 86057-970, Brazil; oba@uel.br

**Keywords:** poultry, necrotic enteritis, predisposing factors, *Clostridium perfringens*, stress

## Abstract

**Simple Summary:**

The ban of antibiotic growth promoters in animal feed increased the number of cases of necrotic enteritis (NE) in broilers, greatly affecting the poultry industry. The induction of experimental NE faces challenges, as it is a multifactorial disease and the pathogenesis is not fully understood, hampering the development of in vivo studies for disease control and prevention strategies. The literature reports several protocols using different factors to assist in NE induction. This study assessed predisposing factors, such as immunosuppression, infection or both, by *Eimeria* spp. in broilers (*n* = 99) fed a wheat-based diet and challenged with three different strains of *Clostridium perfringens* (CP). Under microscopy, *Eimeria* spp. had a negative effect on intestinal morphometry and favored the increase of intraepithelial lymphocytes. However, the macroscopic analysis did not show which factor was more effective in potentiating the lesions, suggesting a synergistic effect between the strain of CP used and the predisposing factors. Therefore, each experimental protocol should first be evaluated for the association of the CP strain with the predisposing factors.

**Abstract:**

*Clostridium perfringens* is the etiological agent of NE, a disease that greatly affects the poultry industry. Experiments on the induction of NE are difficult to carry out, as it is a multifactorial disease, and thus different predisposing factors have been used. This study evaluated the effect of the Gumboro disease vaccine virus vaccine (IBDV-vac) associated or not with infection by *Eimeria* spp. in broilers, as a predisposing factor for NE. Broilers (*n* = 99) were divided into groups (11) challenged with IBDV-vac, *Eimeria* spp. CP type G (CP13, CP14 and CP03) or both. The macroscopic evaluation revealed that the highest average (3.45) of injury occurred for the CP13 + IBDV-vac group. The microscopic analysis showed that *Eimeria* spp. increased the population of intraepithelial lymphocytes and reduced the villus/crypt ratio in duodenum and jejunum when associated with CP13 or CP14. There was a synergistic effect between the CP strain used and the predisposing factors; nevertheless, it was not clear which was the most effective predisposing factor to potentiate the lesions, suggesting that the association of the strain with the factors should first be evaluated for each experimental protocol.

## 1. Introduction

For many years, antimicrobial growth promoters (AGPs) added to feed were used to control necrotic enteritis (NE) in poultry. However, concerns about the spread of multidrug-resistant bacteria led to banning of AGPs, which resulted in an increase of NE cases [1]. NE is a bacterial disease caused by *Clostridium perfringens* (CP) type G, which inflicts losses to the poultry industry in the amount of roughly USD 6 billion per year worldwide [2,3,4]. These losses are mainly due to reduced zootechnical performance with a decrease of up to 12% in body weight and an increase of approximately 11% in the feed conversion rate in relation to healthy birds [5].

Toxins and virulence factors intensify NE, such as enterotoxin (CPE), necrotic enteritis-like toxin B-like (NetB) toxin, and b2-toxin [6]. The new toxigenic classification of CP is based on the toxin production and the pathological condition, thus the type G group, producer of α and NetB toxins, is responsible for NE [7,8]. NE can have clinical or subclinical manifestations, however, there is a predominance of subclinical conditions, reducing zootechnical performance in poultry [9] and increased condemnation for cholangiohepatitis [10,11].

As NE is a multifactorial disease, different protocols have been described in the literature. Nevertheless, there is evident difficulty in inducing the experimental disease, mainly in the clinical form [12]. The use of coccidia infection to promote tissue damage is commonly used [13,14,15]. Other studies also associate immunosuppression with infectious bursal disease virus (IBDV) [16,17], as it makes it difficult to eliminate the infection caused by CP [18]. The supply of diets containing high levels of non-starch polysaccharides with wheat and other grains [19] increase the digesta viscosity and prolong intestinal transit time [20,21], favoring CP growth.

Studies have investigated NE pathogenesis for many years to find prevention and control strategies [22]. However, the essential predisposing factors for the emergence of the disease are not fully understood, due to several variables that can lead to the occurrence of the disease [5]. Several experimental protocols have successfully induced NE in birds; however, the reproducibility of these trials is still an obstacle [23].

In this study, we evaluated three strains of *C. perfringens* type G against infection by *Eimeria* spp. associated or not with IBDV-vac in the induction of NE in broilers.

## 2. Materials and Methods

### 2.1. Bacterial Samples

*Clostridium perfringens* CP13, CP14, and CP03 were isolated from clinical cases of NE. The toxigenic group was classified using polymerase chain reaction (PCR). For that, the CP strains were cultured in BD Difco Brain Heart Infusion (BHI) broth (Crawley, UK) at 37 °C, 18–24 h, and in anaerobic conditions using BD GasPak (Crawley, UK). Then, DNA was extracted using PureLink Genomic DNA Kit Mini Invitrogen (Vilnius, Lithuania), following the manufacturers’ recommendations. The PCR was performed using primers NetB according to Keyburn et al. [24] and the *plc* according to Rood et al. [7]. Amplifications were performed in a Techne thermocycler (Stone, UK) and the amplified products were observed using agarose gel (1.5%) stained with gelred biotium in a Loccus transilluminator.

### 2.2. In Vivo Test

In this study, we used mixed broilers (*n* = 99), Ross 308 lineage (Campinas, Brazil), and one day of age (DA). The animals were housed in experimental cages, and received water and food ad libitum and heating, according to the physiological requirements. At 14 DA, the animals were transferred to cages containing shavings for bedding, decontaminated with paraformaldehyde. The project was approved (Nº 019.2021) by CEUA/UEL. Animals were randomly assigned to 11 groups (Table 1).

The experimental diet for all birds comprised a maize-based diet and soybean meal up to seven days. Next, a wheat-based (62.75%) diet and soybean meal (29.6%) were provided, following the formulation proposed by Du et al. [25].

### 2.3. Inoculum Preparation and Challenge

CP strains were cultured in BHI broth and incubated for 18–24 h at 37 °C under anaerobic conditions using GasPak [26]. Between the 15th and 19th DA, 1 mL (10^7^ CFU/mL) was administered by gavage twice a day to the animals (Table 1). The birds in the negative control group received 1 mL of sterile BHI broth.

At 13 DA, 10 times the dose of the Bio-Coccivet vaccine (Biovet) was administered via gavage to the groups described in Table 1. At 14 DA, the birds (Table 1) received 10 times the dose recommended by the manufacturer, via subcutaneous route, of the Poulvac Magniplex vaccine, Zoetis (São Paulo, Brazil), (IBDV-vac), containing IBDV with attenuation (intermediate plus IBDV vaccine strain).

### 2.4. Intestine Gross Lesion Scoring

On the 20th DA, the birds were euthanized by cervical dislocation and autopsied. The macroscopic appearance of the intestine was blindly evaluated by two experienced pathologists, applying the model proposed by Teirlynck et al. [27].

### 2.5. Quantification of Aerobic and Anaerobic Bacteria

At 20 DA, the liver was collected according to Latorre et al. [28], with some modifications. A portion of the right caudal lobe of the liver was aseptically collected and placed in a sterile bag. Next, BHI broth was added at a 1:10 ratio and then 100 µL of the sample was plated on a spread plate for quantification on MacConkey agar incubated at 37 °C/24 h in aerobic conditions and on Shahidi–Ferguson Perfringens (SFP) agar at 37 °C/24 h under anaerobic conditions. Bacterial translocation was expressed in colony forming units (Log_10_ CFU/g).

### 2.6. Histological Analysis

Intestinal samples (*n* = 6/group) were collected according to the Swiss roll technique and subsequently submerged in buffered formalin (10%) for 24 h, then conditioned in ethanol (70%) until histological preparation, according to Souza et al. [29]. Tissue sections of 5 μm were stained with hematoxylin and eosin (HE) and then we evaluated villus height, crypt depth, small intestine villus/crypt ratio, and intraepithelial lymphocyte (iIEL) count. Alcian Blue staining was also performed to quantify the goblet cells of the ileum. All analyses were performed according to Souza et al. [30].

The microscopic intestinal lesion score analysis was performed only in the groups with CP associated with *Eimeria* spp., due to the damage caused by the oocysts to the avian intestine. Histological changes were evaluated using a lesion score scale, considering the injury intensity as described by Terciolo et al. [31]. The lesion score was established by considering the severity degree (severity factor) and the extent of each lesion (according to intensity or observed frequency, scored from 0 to 3). For each lesion, the extent score was multiplied by the severity factor. The following morphological and lesional criteria were included to the score: flattening of enterocytes, villi atrophy and fusion, interstitial edema, lymphatic vessel dilation, loss of apical enterocytes, cell vacuolation, and necrotic debris.

### 2.7. Statistical Analysis

The data obtained were submitted to analysis of variance (ANOVA) for a randomized block design, with two blocks, 11 groups and six repetitions for histological analysis and up to 9 repetitions for the other analyses, followed by the Scott–Knott at 5% probability. All analyses were performed using RStudio version 2021.09.1-372 (Boston, MA, USA).

## 3. Results

### 3.1. Experimental Infection

The *Clostridium perfringens* samples used in this study were confirmed to be type G, positive for NetB and alpha toxin.

**Figure 1:**
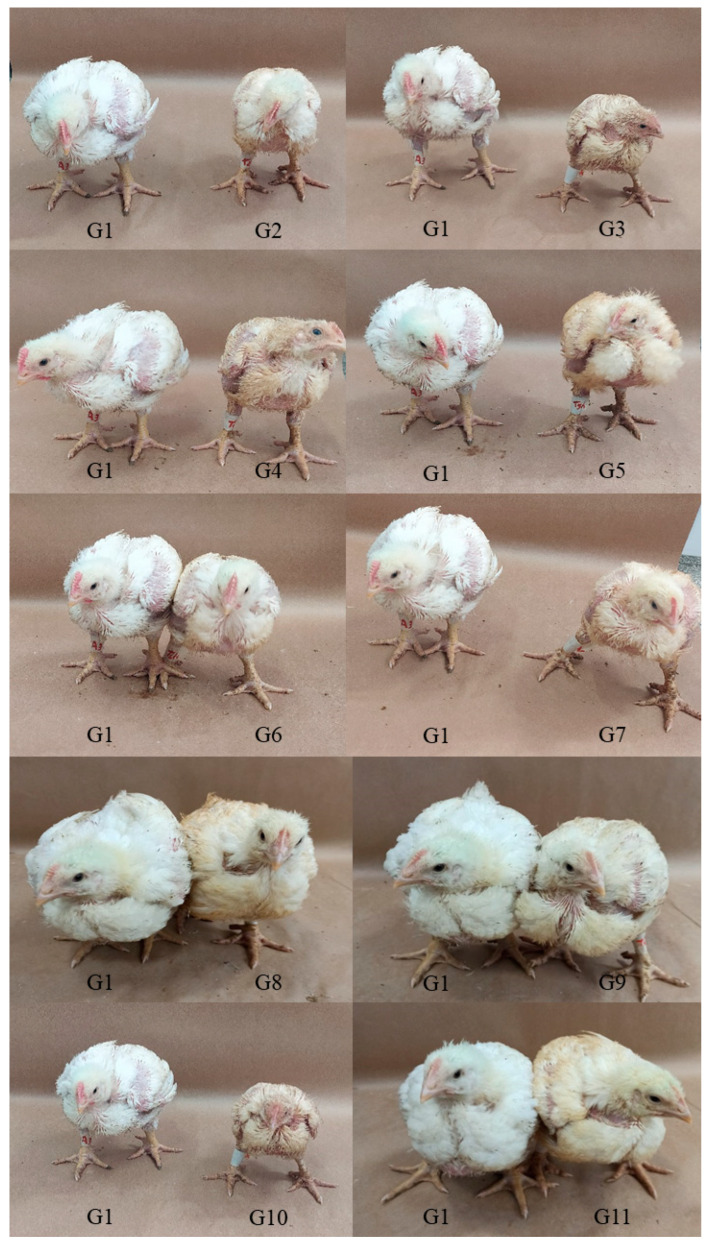
Presence of nonuniformity between the groups in relation to the negative control. G1—negative control; G2—CP13 + *Eimeria* spp.; G3—CP13 + IBDV-vac; G4—CP13 + *Eimeria* spp. + IBDV-vac; G5—CP14 + *Eimeria* spp.; G6—CP14 + IBDV-vac; G7—CP14 + *Eimeria* spp. + IBDV-vac; G8—CP03 + *Eimeria* spp.; G9—CP03 + IBDV-vac; G10—CP03 + *Eimeria* spp. + IBDV-vac; G11—*Eimeria* spp.

From the second day of challenge onward, agglomeration of birds in the cages, diarrhea, hyporexia, apathy, and depression was observed, mainly in groups G4 (CP13 + IBDV-vac + *Eimeria* spp.), G7 (CP14 + IBDV-vac + *Eimeria* spp.), and G10 (CP03 + IBDV-vac + *Eimeria* spp.). Nonuniformity was also observed in challenged birds in relation to animals in the negative control group (Figure 1). This characteristic was more evident in birds from G3 (CP13 + IBDV-vac) and G10 (CP03 + IBDV-vac + *Eimeria* spp.). Despite all care, two birds from the G7 and three G1 group died during the trial.

### 3.2. Evaluation of Gross Lesions of the Intestine

Table 2 presents the results of the gross intestinal lesions.

The gross pathology analysis to evaluate the exclusive effect of the *Eimeria* spp. between treatments did showed that groups G2 (CP13 + *Eimeria* spp.) (2.89) and G5 (CP14 + *Eimeria* spp.) (2.78) significantly differed (*p* ≤ 0.05) from G8 (CP03 + *Eimeria* spp.), that had the highest average (3.34) (Table 2). When analyzing the IBDV-vac variable, the G3 (CP13 + IBDV-vac) and G6 (CP14 + IBDV-vac) groups had the highest averages, 3.45 and 2.56, respectively, differing significantly (*p* ≤ 0.05) from the G9 (CP03 + IBDV-vac) with an average of 2.23 (Table 2).

When evaluating the effect of CP strains, the G2 (CP13 + *Eimeria* spp.) and G3 (CP13 + IBDV-vac) groups had higher lesion averages, 2.89 and 3.45, respectively, and were significantly different (*p* ≤ 0.05) from the CP13 + *Eimeria* spp. + IBDV-vac (1.67). In the groups challenged with CP14, the highest average lesion found was in G5 (CP14 + *Eimeria* spp.) (2.78) and G6 (CP14 + IBDV-vac) (2.56), which differed from G7 (CP14 + *Eimeria* spp. + IBDV-vac) (1.78). In the CP03 challenge, the highest injury averages were found in the CP03 + *Eimeria* spp. (3.34) and CP03 + *Eimeria* spp. + IBDV-vac (2.43), which differed significantly (*p* ≤ 0.05) from CP03 + IBDV-vac (2.23), a behavior different from that observed for strains CP13 and CP14.

### 3.3. Quantification of Aerobic and Anaerobic Bacteria in the Liver

Table 3 shows the results of the average quantification of aerobic and anaerobic bacteria in the livers of birds, referring to increased permeability and breakdown of the intestinal barrier.

Regarding anaerobic bacteria, a greater bacterial translocation was observed in the groups that received CP13, CP14, and CP03 associated with *Eimeria* spp. + IBDV-vac, suggesting a positive influence of these factors for bacterial migration.

### 3.4. Histological Evaluation of the Intestine

**Figure 2:**
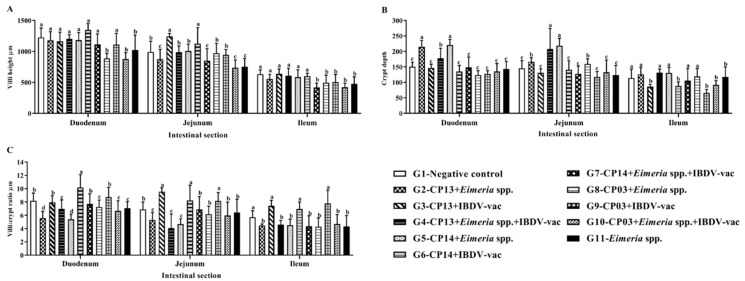
Mean villus height, crypt depth, and villus/crypt ratio of the different groups. ^a,b,c^ Different letters in the column indicate a significant difference (*p* ≤ 0.05) between groups. Scott–Knott test at 5% significance level.

The results obtained in the morphometric analysis (Figure 2) showed a significant decrease (*p* ≤ 0.05) in villus height in groups G8 (CP03 + *Eimeria* spp.) and G10 (CP03 + *Eimeria* spp. + IBDV-vac) in the duodenum in relation to the other groups, however, there was no difference from the G11 group (*Eimeria* spp.). The lowest mean villus height in the jejunum and ileum were observed in the G10 (CP03 + *Eimeria* spp. + IBDV-vac) and G7 (CP14 + *Eimeria* spp. + IBDV-vac) groups, respectively.

Regarding the crypts, in the duodenum, greater depth was found in birds challenged with CP13 (G2) and CP14 (G5) associated with *Eimeria* spp., in relation to the negative control (G1). In the jejunum, the G4 group (CP13 + *Eimeria* spp. + IBDV-vac) and G5 group (CP14 + *Eimeria* spp.) showed more pronounced crypt depths, significantly different from the others (*p* ≤ 0.05).

Figure 2 shows that groups CP13 (G2) and CP14 (G5) associated with *Eimeria* spp. had a lower villus/crypt (V:C) ratio in the duodenum, differing significantly (*p* ≤ 0.05) from the control. In the jejunum, the G2 (CP13 + *Eimeria* spp.), G4 (CP13 + *Eimeria* spp. + IBDV-vac), and G5 (CP14 + *Eimeria* spp.) groups differed significantly from the control group (*p* ≤ 0.05).

The population of intraepithelial lymphocytes showed a significant difference (*p* ≤ 0.05) only in the duodenum segment (Table 4). Groups G5 (CP14 + *Eimeria* spp.), G8 (CP03 + *Eimeria* spp.), and G11 (*Eimeria* spp.) had the highest means and did not differ from each other, differing significantly (*p* ≤ 0.05) from G1. The number of goblet cells in the ileum showed no significant difference between groups (*p* > 0.05).

**Figure 3:**
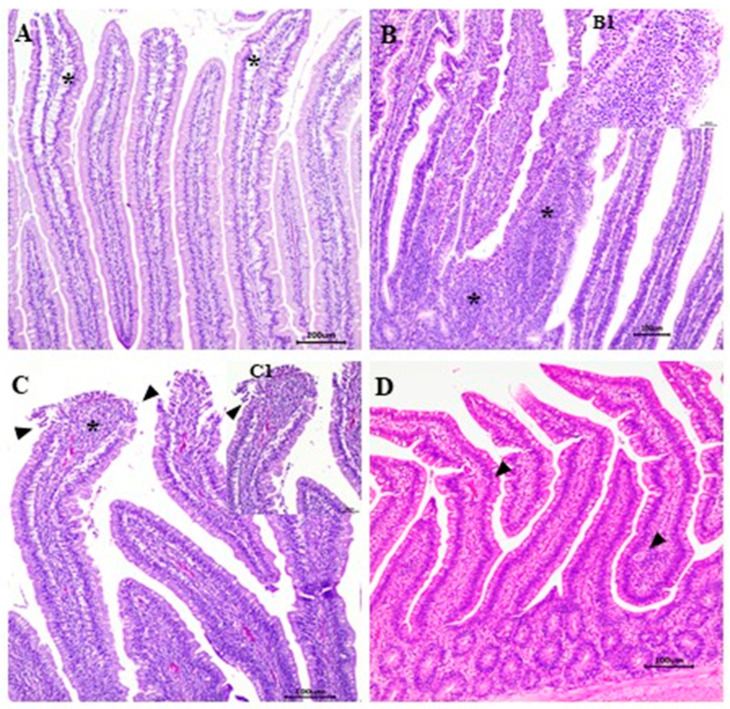
Changes observed in the microscopic score. (**A**) Duodenum, G6 (CP14 + IBDV-vac), interstitial edema (*), bar 100 µm, HE. (**B**) Jejunum, G11 (*Eimeria* spp.), moderate presence of inflammatory infiltrate (*), bar 100 µL, HE. (**B1**) Insert—inflammatory infiltrate, bar 50 µm, HE. (**C**) Duodenum, G11 (*Eimeria* spp.) villus apical necrosis, moderate presence of inflammatory infiltrate (*) bar 100 µL, HE. (**C1**) Insert—villus apical necrosis (▼) (bar 50 µm, HE. (**D**) Jejunum, G2 (CP13 + *Eimeria* spp.), *Eimeria* spp. (▼), bar 100 µL, HE.

In the microscopic score analysis, a significant difference (*p* ≤ 0.05) was observed only in the jejunum (*p* ≤ 0.05). The negative control had the lowest mean (1.2), different from the other groups, with *Eimeria* spp. presenting the highest mean (8.67), not different from CP13 + *Eimeria* spp. (5.83) and CP03 + *Eimeria* spp. (8.17). (Table 5). The main changes observed in the microscopic score were edema, inflammatory infiltrate (Figure 3), and congestion.

## 4. Discussion

NE triggers an inflammatory response in the intestines of birds [32] and dysbiosis [33,34], causing energy imbalance and alteration in skeletal muscle growth [35].

Studies point to difficulties in inducing experimental NE infection in chickens [12,36]. The challenge using CP exclusively, without association with predisposing factors, does not allow the pathogen establishment in the intestine, expression of clinical signs, as well as significant changes in the microbiota of birds [37,38]. Thus, several factors are used to predispose to the disease development, such as the administration of *Eimeria* spp., immunosuppression, diets with a high concentration of non-starch polysaccharides [12] and heat stress [18] which must be combined with the challenge with virulent CP for an efficient development of the disease experimentally [39].

In this study, when we associated the different strains of CP with the predisposing factors *Eimeria* spp. + IBDV-vac, we observed the expression of mild to moderate clinical signs, such as lethargy, apathy, hyporexia, but no mortality. Signs became evident from the second day of bacterial challenge, corroborating Latorre et al. [28]. The authors reported similar clinical signs and no mortality during the NE induction experiment in birds. In this study, the signs were more evident in the G4 (CP13 + *Eimeria* spp. + IBDV-vac), G7 (CP14 + *Eimeria* spp. + IBDV-vac), and G10 (CP03 + *Eimeria* spp. + IBDV-vac) groups, suggesting that the association of *Eimeria* spp. + IBDV-vac favored the expression of clinical signs in challenged birds, regardless of the strain used, different from what was found in relation to intestinal lesions and bacterial translocation.

The experimental challenge of this study induced a subclinical necrotic enteritis model, corroborating previous studies [40,41,42,43].

Experimentally, it is not always possible to observe macroscopic lesions of NE [44], the induction protocol influences the inflammation, and the severity of intestinal lesions [45]. In a similar way, the CP strain plays an important role in the intensity of injuries and the severity of the disease [23].

The macroscopic intestinal lesions observed were predominantly mild and more expressive in the duodenum and jejunum, corroborating Liu et al. [46]. According to Huang et al. [47] NE lesion scores are higher after one day of CP challenge and the lesions are mild at seven days. After infection with CP, the organism modulates strategies, upregulating protein components, to maintain intestinal integrity and reduce the damage caused by the bacteria [35].

In this study, no significant differences (*p* > 0.05) were observed between the groups (G1–G11) when we fixed the variable *Eimeria* spp. and we compared the effect of different CP strains, indicating that the result was similar when associating *Eimeria* spp., regardless of the CP strain used (Table 2). When fixing the IBDV-vac variable, we found a difference in the gross lesion score between the strains used, with emphasis on CP13 (3.45) and CP14 (2.56), which presented the highest means, indicating a synergistic effect of the strain with the IBDV-vac variable (Table 2).

When evaluating the effect of the variables (*Eimeria* spp., IBDV-vac, and *Eimeria* spp. + IBDV-vac) against each strain studied, we observed that the factors *Eimeria* spp. or IBDV-vac associated with CP13 and CP14 strains provided a higher degree of intestinal lesion. However, the most expressive lesion degree, when using CP03, was found when associating *Eimeria* spp. or *Eimeria* spp. + IBDV-vac (Table 2), suggesting a factor that intensifies the degree of lesion for each strain. The highest means of intestinal injury were observed in G3 (CP13 + IBDV-vac) and G8 (CP03 + *Eimeria* spp.).

The exclusive use of IBDV-vac does not seem to potentiate the degree of intestinal lesion by the CP03 strain; however, the opposite was observed by the CP13 and CP14 strains (Table 2). These data corroborate Chalmers et al. [48] who observed a different behavior when evaluating five CP strains and only one was capable of inducing a condition compatible with NE.

In this study, the *Eimeria* spp. + IBDV-vac combination positively favored the CP13, CP14, and CP03 strains in terms of bacterial translocation (Table 3). The presence of bacteria in the liver plays a significant role in the intestinal health of birds [27], as *Clostridium perfringens* infection decreases the expression of tight junction proteins [49] and CP toxins promote increased intestinal permeability and consequently advantage bacterial translocation [50] and passage of endotoxins through the intestinal tract mucosa to extra-intestinal sites, such as the liver [51]. Latorre et al. [28] observed a higher concentration of aerobic and anaerobic bacteria in the livers of birds challenged with CP compared to the control group, partially corroborating our study, in which we found a significant difference (*p* ≤ 0.05) from the control group, only in the count of anaerobic bacteria.

The presence of bacteria in the livers of birds in the control group (G1) may be related to the dysbiosis caused by the wheat-based diet, since this component has a high content of non-starch polysaccharides [47,52] and favors a reduction in the passage rate of intestinal contents, an increase in viscosity [53], in addition to providing complex carbohydrates for CP growth [54]. Redondo et al. [55] observed lesions in birds did not challenge with CP, but fed with a high-protein diet, suggesting an increase in the population of commensal CP, initiating the natural NE infection [48].

The height of the villi and the depth of the crypts have a direct correlation with intestinal integrity [56]. It is known that intestinal villi increase the contact surface with the content in the lumen, allowing for greater absorption of nutrients [57]. The challenge with CP can change the morphology and reduce the height of the intestinal villi [49], while causing lesions at the apex or even throughout the villi [41], which implies lower absorption of nutrients, compromising the growth of poultry chickens.

In the duodenum, the groups G8 (CP03 + *Eimeria* spp.) and G10 (CP03 + *Eimeria* spp. + IBDV-vac) promoted a significant decrease in the height of the intestinal villi (Figure 2); however, the height in these groups did not differ significantly (*p* > 0.05) from that in the G11 group (*Eimeria* spp.). In the jejunum, groups G2 (CP13 + *Eimeria* spp.), G7 (CP14 + *Eimeria* spp. + IBDV-vac), and G10 (CP03 + *Eimeria* spp. + IBDV-vac) had the lowest villus heights (Figure 2); however, the height in these groups did not differ from that in the G11 group (*Eimeria* spp.), indicating a possible effect of infection by *Eimeria* spp. on villus height, since coccidia compromise intestinal integrity and increase mucus production and plasma extravasation into the lumen, which are a nutrient source for CP growth [58], potentiating the NE lesions [45].

CP infections show an increase at the depth of the crypts and a decrease in the villus/crypt ratio, increasing the metabolic expenses of the intestinal epithelium turnover and reducing the capacity of nutrient absorption by the intestine [59].

In this study, in the duodenum, the depth of the crypts was more pronounced in groups G2 (CP13 + *Eimeria* spp.) and G5 (CP14 + *Eimeria* spp.) and the V:C ratio was lower in these groups. In the jejunum, groups G4 (CP13 + *Eimeria* spp. + IBDV-vac) and G5 (CP14 + *Eimeria* spp.) showed greater depth of crypts and lower V:C ratio, differing significantly (*p* ≤ 0.05) from group G11 (*Eimeria* spp.). Conversely, in the ileum, no significant difference (*p* > 0.05) was observed between groups G5 and G11, indicating greater CP influence in the duodenum and jejunum segments (Figure 2). These results corroborate M’Sadeq et al. [60], who observed reduced intestinal villi by challenge with CP and *Eimeria* spp., a lower V:C ratio, and an increase in the crypt depth. Golder et al. [61] found deeper crypts in the group with *Eimeria* spp. + CP compared to the negative control group.

The release of pro-inflammatory cytokines in cases of subclinical NE mobilizes leukocytes to the inflammation site [59]. Thus, intestinal intraepithelial lymphocytes play an important role in the protection against intestinal infection and act as modulators in antigen presentation [62].

This study showed that the use of *Eimeria* spp. (G11) alone or associated with CP14 (G5) and CP03 (G8), stimulated an increase in the population of iIEL in the duodenum (Table 4). Ruhnke et al. [63] observed an increase in the number of intraepithelial lymphocytes in the intestine of broilers challenged with CP and *Eimeria* spp. On the other hand, we observed that the groups that received IBDV-vac (G3, G4, G6, G7, G9 and G10) had a lower iIEL count in the duodenum and did not differ from the negative control (Table 4). This behavior can be explained by the damage that IBDV-vac causes to the immune system, which leads to a significant decrease in the lymphocyte population, affecting the development, maturation, and induction to apoptosis of lymphocytes [64].

The microscopic lesion scoring system supports the assessment of subtle intestinal damage and can thus be used as an alternative to verify differences between groups, even without severe NE lesions in the intestine [65].

In this study, a significant difference (*p* ≤ 0.05) was observed only in the jejunum and the group containing only *Eimeria* spp. did not differentiate from CP13 + *Eimeria* spp. and CP03 + *Eimeria* spp. (Table 5), suggesting that the lesions in the microscopic lesion score are related to the administration of *Eimeria* spp. in addition to the presence of oocysts of *Eimeria* spp. predominantly in the jejunum, reinforcing the hypothesis that *Eimeria* spp. influenced the increase in microscopic lesions.

The main microscopic changes found were edema, congestion, and inflammatory infiltrate (Figure 3). Sanches et al. [56] did not observe macroscopic NE lesions in birds challenged with CP using 15 times the dose of *Eimeria* spp. vaccine. In the microscopic evaluation, however, the authors identified congestion, infiltration of inflammatory cells into the lamina propria, and inflammatory cells in the epithelium, similar to the results found in our study.

Different studies [16,18,66] indicate that a diet with a high concentration of non-starch polysaccharides, environmental stress, immunosuppressive diseases, and infection by *Eimeria* spp. are important predisposing factors for the development of NE in broilers. However, the pathogenesis of the disease is still not completely understood [53], therefore, investigations of NE induction associated with predisposing factors could help clarify the pathogenesis and support strategies for disease control [38].

## 5. Conclusions

This study showed a possible association between the CP strain used and the predisposing factors. Nevertheless, it was not clear which predisposing factor is more effective in potentiating the lesions, suggesting the association of the CP strain to predisposing factors for each experimental protocol.

## Figures and Tables

**Table 1:** Description of experimental groups.

Groups	Description	*n*
G1	Negative control	9
G2	CP * 13 + *Eimeria* spp.	9
G3	CP13 + IBDV-vac **	9
G4	CP13 + *Eimeria* spp. + IBDV-vac	9
G5	CP14 + *Eimeria* spp.	9
G6	CP14 + IBDV-vac	9
G7	CP14 + *Eimeria* spp. + IBDV-vac	9
G8	CP03 + *Eimeria* spp.	9
G9	CP03 + IBDV-vac	9
G10	CP03 + *Eimeria* spp. + IBDV-vac	9
G11	*Eimeria* spp.	9
Total		99

* CP—*Clostridium perfringens*, ** IBDV-vac—infectious bursal disease virus vaccine.

**Table 2:** Means of the gross lesion score of the different groups.

Groups	Means
G1	2.75 ± 1.48 ^a^
G2	2.89 ± 1.57 ^a^
G3	3.45 ± 0.51 ^a^
G4	1.67 ± 0.68 ^b^
G5	2.78 ± 0.94 ^a^
G6	2.56 ± 1.54 ^a^
G7	1.78 ± 0.94 ^b^
G8	3.34 ± 1.08 ^a^
G9	2.23 ± 0.43 ^b^
G10	2.43 ± 2.87 ^a^
G11	2.56 ± 1.09 ^a^

G1—negative control; G2—CP13 + *Eimeria* spp.; G3—CP13 + IBDV-vac; G4—CP13 + *Eimeria* spp. + IBDV-vac; G5—CP14 + *Eimeria* spp.; G6—CP14 + IBDV-vac; G7—CP14 + *Eimeria* spp. + IBDV-vac; G8—CP03 + *Eimeria* spp.; G9—CP03 + IBDV-vac; G10—CP03 + *Eimeria* spp. + IBDV-vac; G11—*Eimeria* spp. ^a,b^ Different letters in the column indicate a significant difference (*p* ≤ 0.05) between groups. Scott–Knott test at 5% significance level.

**Table 3:** Means (Log_10_ CFU/g) of bacterial quantification in aerobic and anaerobic present in the liver of birds of different groups.

Groups	Aerobic	Anaerobic
G1	0.78 ± 0.91	0.75 ± 1.20 ^b^
G2	1.15 ± 0.93	2.25 ± 0.52 ^a^
G3	0.16 ± 0.49	1.23 ± 1.18 ^b^
G4	1.06 ± 0.99	2.61 ± 0.32 ^a^
G5	0.461 ± 0.94	2.26 ± 0.38 ^a^
G6	0.86 ± 1.07	2.38 ± 0.49 ^a^
G7	0.58 ± 0.95	2.65 ± 0.71 ^a^
G8	0.57 ± 0.92	1.44 ± 1.58 ^b^
G9	0.22 ± 0.44	0.88 ± 1.53 ^b^
G10	0.71 ± 1.22	2.46 ± 0.81 ^a^
G11	0.51 ± 0.61	0.99 ± 1.23 ^b^

G1—negative control; G2—CP13 + *Eimeria* spp.; G3—CP13 + IBDV-vac; G4—CP13 + *Eimeria* spp. + IBDV-vac; G5—CP14 + *Eimeria* spp.; G6—CP14 + IBDV-vac; G7—CP14 + *Eimeria* spp. + IBDV-vac; G8—CP03 + *Eimeria* spp.; G9—CP03 + IBDV-vac; G10—CP03 + *Eimeria* spp. + IBDV-vac; G11—*Eimeria* spp. ^a,b^ Different letters in the column indicate a significant difference (*p* ≤ 0.05) between groups. Scott–Knott test at 5% significance level.

**Table 4:** Means of small intestine intraepithelial lymphocyte (iIEL) count and ileal goblet cell count.

Groups	Duodenum	Jejunum	Ileum	
	iIEL	iIEL	iIEL	Goblet Cells
G1	22.6 ± 6.04 ^b^	18.32 ± 3.68	9.38 ± 12.74	96.65 ± 17.98
G2	26.42 ± 6.36 ^b^	25.11 ± 13.09	6.11 ± 5.13	97.53 ± 16.87
G3	21.54 ± 5.02 ^b^	31.83 ± 5.64	7.26 ± 1.37	90.02 ± 20.13
G4	24.76 ± 8.04 ^b^	24.08 ± 9.45	6.96 ± 2.99	123.08 ± 8.09
G5	33.56 ± 9.35 ^a^	25.07 ± 11.23	5.51 ± 2.67	100.89 ± 13.71
G6	28.90 ± 5.73 ^b^	26.19 ± 5.99	10.22 ± 4.80	100.22 ± 21.71
G7	27.12 ± 9.70 ^b^	32.89 ± 17.20	12.05 ± 9.72	88.57 ± 12.38
G8	38.46 ± 12.76 ^a^	35 ± 10.76	9.85 ± 4.86	104.06 ± 10.14
G9	28.34 ± 11.65 ^b^	33.17 ± 9.38	8.65 ± 3.81	107.95 ± 24.52
G10	23.79 ± 12.86 ^b^	36.24 ± 16.50	9.54 ± 6.01	102.55 ± 18.33
G11	39.54 ± 8.12 ^a^	31.44 ± 8.32	13.32 ± 8.52	114.8 ± 15.26

G1—negative control; G2—CP13 + *Eimeria* spp.; G3—CP13 + IBDV-vac; G4—CP13 + *Eimeria* spp. + IBDV-vac; G5—CP14 + *Eimeria* spp.; G6—CP14 + IBDV-vac; G7—CP14 + *Eimeria* spp. + IBDV-vac; G8—CP03 + *Eimeria* spp.; G9—CP03 + IBDV-vac; G10—CP03 + *Eimeria* spp. + IBDV-vac; G11—*Eimeria* spp. ^a,b^ Different letters in the column indicate a significant difference (*p* ≤ 0.05) between groups. Scott–Knott test at 5% significance level. Means (μm).

**Table 5:** Mean scores of microscopic lesions of the duodenum and jejunum of the groups administered *Eimeria* spp.

Groups	Duodenum	Jejunum
G1	1.20 ± 0.45	1.2 ± 0.83 ^c^
G2	3.17 ± 1.33	5.83 ± 2.79 ^a^
G5	2.50 ± 2.07	4.5 ± 1.52 ^b^
G8	2.83 ± 1.47	8.17 ± 2.79 ^a^
G11	2.67 ± 1.03	8.67 ± 3.20 ^a^

G1—negative control; G2—CP13 + *Eimeria* spp.; G5—CP14 + *Eimeria* spp.; G8–CP03 + *Eimeria* spp.; G11—*Eimeria* spp. ^a,b,c^ Different letters in the column indicate a significant difference (*p* ≤ 0.05) between groups. Scott–Knott test at 5% significance level.

## Data Availability

The data presented in this study are available upon request from the corresponding author.
